# Medicinal Plants and Natural Active Compounds for Diabetes and/or Obesity Treatment

**DOI:** 10.1155/2015/469762

**Published:** 2015-10-19

**Authors:** Hilal Zaid, Bashar Saad, Abbas A. Mahdi, Akhilesh K. Tamrakar, Pierre S. Haddad, Fatma U. Afifi

**Affiliations:** ^1^Qasemi Research Center, Al-Qasemi Academy, P.O. Box 124, 30100 Baqa El-Gharbia, Israel; ^2^Faculty of Arts and Sciences, Arab American University-Jenin, P.O. Box 240, Jenin, State of Palestine; ^3^Department of Biochemistry, King George's Medical University, Lucknow 226003, India; ^4^Division of Biochemistry, CSIR-Central Drug Research Institute, Lucknow 226031, India; ^5^Department of Pharmacology, University of Montreal, Montreal, QC, Canada H3C 3J7; ^6^Faculty of Pharmacy, University of Jordan, Amman 11942, Jordan

Diabetes has been recognized since ancient times, and its main symptoms were known by the increased thirst, frequent urination, and tiredness. Obesity is one of the major risk factors for a number of chronic diseases, especially type 2 diabetes (T2D), leading to increase in healthcare costs and decrease in life expectancy. Free fatty acids (FFA) represent a crucial link between obesity, inflammation, and insulin resistance and, as such, reduction in elevated plasma FFA should be an important therapeutic target in obesity and T2D. According to the World Health Organization (WHO), 35% of adults aged 20 and over were overweight in 2008, and 11% were obese. Moreover, T2D prevalence has increased from less than 10% in 1980 to more than 30% nowadays [1].

There are several types of glucose-lowering drugs [2], including insulin sensitizers (biguanides, metformin, and thiazolidinediones), insulin secretagogues (sulfonylureas, meglitinides), and *α*-glucosidase inhibitors (miglitol, acarbose). Most glucose-lowering drugs, however, may have side effects, such as severe hypoglycemia, idiosyncratic liver cell injury, lactic acidosis, permanent neurological deficit, digestive discomfort, headache, and dizziness [3, 4]. As a result, researchers are interested in finding more efficient medicines, with less side effects. Medicinal plant drug discovery provides important leads against various pharmacological targets including T2D and obesity.

With the dramatically increasing prevalence of obesity and T2D worldwide, there is an urgent need for new strategies to combat the growing epidemic of these metabolic diseases. Diet is an essential factor affecting the development of obesity and T2D and it can either prevent or accelerate metabolic diseases. In searching for preventative and therapeutic strategies, it is therefore advantageous to consider the potential of certain medicinal plants as well as herbal-based foods and their bioactive compounds to prevent/treat the pathogenic processes associated with these diseases. To date, the concept of antidiabetic and antiobesity medicinal plants is highlighted in textbooks and pharmaceutical pamphlets and has been reported in thousands of scientific publications. Yet, most of these publications report the activity of a crude extract without testing its chemical composition or identifying the active compound(s) or even its mechanism of action. We believe that natural novel drugs are now more achievable due to modern techniques for separation, structure elucidation, screening, and bio- and chemoinformatics. But whatever approach is used, the medicinal plant efficacy will be based on* in vitro* or* in vivo* bioassays.

This special issue on medicinal plants for the treatment of diabetes and obesity is a bird's eye view on up-to-date knowledge of promising traditional medicines and their active ingredients efficacy and mechanisms of action in treating obesity and T2D. Nine selected papers for publication in the present issue summarize the most recent knowledge and techniques to evaluate the medicinal plants and active compounds for their antidiabetic, antiobesity, and antioxidant activity* in vitro* and* in vivo*. Manuscripts in this special issue cover several aspects of recent developments in the fields of (a) medicinal plants, buckwheat honey, and natural compounds preventing metabolic disorders (including T2D)* in vivo* and* in vitro*; (b) antioxidant and anti-inflammatory natural products; (c) phenolic compounds that show adipogenesis activity* in vitro*; (d) herbal pharmacotherapy and phytochemical studies* in vitro* and* in situ*; (e) examining the reliability of potential antioxidant substance based on the selected assays; (f) studies involving toxicology and pharmacological mechanisms of action of medicinal plants used* in vivo* and* in vitro*.
